# Assessment of Visual and Brainstem Auditory Evoked Potentials in Patients with Hashimoto's Thyroiditis

**DOI:** 10.1155/2021/3258942

**Published:** 2021-03-03

**Authors:** Marta Waliszewska-Prosół, Maria Ejma

**Affiliations:** Department of Neurology, Wrocław Medical University, Wrocław, Poland

## Abstract

**Background:**

The present study was aimed to evaluate parameters of visual and brainstem auditory evoked potentials (VEP, BAEP) in euthyreotic Hashimoto's thyroiditis (HT) patients without central nervous system involvement.

**Methods:**

100 HT patients (92 women, 8 men), mean age 46.9 years, and 50 healthy controls. They underwent a neurological examination, thyroid hormone levels, thyroid autoantibody titers, and brain imaging. Latencies and amplitudes of the N75, P100, and N145 component of VEP and the I-V components of BAEP were analyzed.

**Results:**

The neurological examination revealed in 31 patients signs of increased neurovegetative excitability. Brain resonance imaging showed no abnormalities in HT patients. The mean P100, relative P100, and N145 VEP latencies were significantly longer, and P100 amplitude significantly higher in HT patients than the controls. HT patients also had a longer mean wave BAEP V latency and mean wave III-V and I-V interpeak latencies, and significantly lower mean wave I and V amplitudes. Abnormal VEP and BAEP were recorded in 34% of the patients. There were no statistically significant correlations between the mean VEP parameters and thyroid profile and the applied dose of L-thyroxine. There was a relationship between the level of TSH and the wave BAEP III-V interpeak latency.

**Conclusions:**

There were changes in the brain's bioelectrical activity in one-third of the patients with HT without nervous system involvement. The increased amplitude of the VEP may indicate increased cerebral cortex activity. Disorders of the brain's bioelectrical activity in the course of HT may be associated with an autoimmune process.

## 1. Introduction

Thyroid hormones play a key role in the development and proper functioning of the nervous system, especially its central part. Due to the broad profile of thyroid hormones on the central nervous system (CNS), symptoms of neurological deficit may occur in hypothyroidism as well as in hyperthyroidism [1–3]. In recent years, attention has also been paid to the possibility of damage to the CNS in the course of autoimmune thyroid disease in people with balanced function of this gland, who remain in the euthyroid stage for many years [2, 4]. Hashimoto's thyroiditis (HT) is the most common endocrine and autoimmune disorder and the most common cause of primary hypothyroidism in both children and adults [1, 5].

The course of HT memory and concentration difficulties is often observed. Patients may experience excessive irritability or heaviness, sleep disorders, anxiety, and depression [1, 3, 6]. Myopathy, neuropathy, ataxia, and encephalopathy are often diagnosed with them [2, 7]. The cause of these symptoms has not yet been identified, and the pathomechanism of these changes is still under investigation.

In recent years, attention has been paid to the impact of hormone-compensated autoimmune thyroiditis on CNS function and the possibility of developing severe neurological complications in euthyroid patients. The severest of these is Hashimoto encephalopathy (EH) or SREAT (S-steroid responsive, E-encephalopathy, A-associated, T-autoimmune thyroiditis) [3, 7–10]. The causes of these disorders are unknown. Morphological and histopathological studies in patients diagnosed with EH showed the presence of chronic, limited inflammatory changes in the cortex and meninges, which were defined by the term NAIM (nonvasculitis autoimmune inflammatory meningoencephalitis) [11]. Some autopsy studies suggest that EH may be associated with lymphocytic infiltration or vasculitis in the brainstem and gray matter [12]. As a causative agent, direct toxic effects of TRH on nerve cells or inflammatory changes of the brain and spinal cord in the course of demyelination were considered. It has also been suggested that EH may be a consequence of a generalized decrease in cerebral perfusion or cytotoxic brainstem edema [2, 13]. It is suspected that the deposition of immune complexes may lead to changes in the central nervous system. This is supported by the results of neuroimaging studies showing the presence of vascular inflammatory changes and cytotoxic edema of the brain in patients with hormonally compensated Hashimoto's disease [2, 11, 14, 15].

The evoked potentials (EP) study has become one of the particularly useful electrophysiological methods used in the diagnosis of functional CNS disorders. Unlike imaging techniques, which only reveal anatomical and structural changes, EPs allow assessment of the integrity and functional activity of the nerve pathways. They are particularly helpful in detecting clinically silent disorders, locating lesions, confirming questionable and ambiguous changes, as well as monitoring the course of some neurological diseases. The main advantages of EP testing are its sensitivity, repeatability, noninvasiveness, and easy way of performing [16]. EP studies have not been conducted in a large group of HT patients, especially in cases with compensated thyroid function. The methodology of these studies was different, and the study groups were most often heterogeneous and included small groups of patients with different thyroid diseases.

Our study was aimed to evaluate brain bioelectrical activity changes in HT patients using visual and brainstem evoked potentials and to correlate EP parameters with clinical data.

## 2. Materials

The study comprised 100 patients (92 women and 8 men, aged 20-68 years, mean 46.9) who met the criteria for the diagnosis of HT [1]. All the patients underwent a thyroid ultrasound examination and were evaluated by an endocrinologist. All patients had compensated thyroid function (normal TSH level) and were treated with levothyroxine.

The exclusion criteria included the following:

- Neurological, systemic, toxic, traumatic and other autoimmune, endocrinopathy, and metabolic diseases (e.g., diabetes, cardiovascular diseases)
- Chronic use of CNS-active medicines (e.g., antiepileptic, neuroleptics, psychostimulants, analgesics, steroids, sedatives, and hypnotics)
- Significant damage to the eye (visual acuity including spectacle correction was determined at 0.9 to 1.0)
- Significant damage to the hearing organ, previous ear diseases - inflammatory, proliferative, traumatic, toxic (e.g. the use of aminoglycosides) and a history of tinnitus, transient balance disorders and dizziness
- Auditory thresholds above 25 dB NA in the mean frequencies from 250 to 2000 Hz
- Underweight (BMI <18.5 kg/m^2^) or obesity with BMI ≥35 kg/m^2^

The control group (CG) consisted of 50 healthy volunteers, who were matched for age and gender to the HT patients (44 women, 6 men, aged 20-68 years, mean 46.3). The neurological and EP protocol as well as the exclusion criteria was the same as in the HT group. In the CG, the presence of thyroid disease and elevated thyroid antibody titers was excluded.

All the subjects gave their informed consent to participate in the study, and the project was approved by the Commission of Bioethics at the Wroclaw Medical University (number of permission: KB-313/2013).

## 3. Methods

The patients underwent a neurological examination and screening tests for cognitive impairment—Montreal Cognitive Assessment (MoCA) and Clock Drawing Test (CDT). All patients underwent brain magnetic resonance imaging (MRI). Laboratory tests included serum concentration of thyrotropin (TSH), free triiodothyronine (fT3), free thyroxine (fT4), and serum autoantibodies against thyroid peroxidase (anti-TPO) and thyroglobulin (anti-TG) titers. Parathyroid hormone levels were measured in patients with tetany symptoms.

The procedures of visual and brainstem auditory evoked potentials were conducted according to the International Federation of Clinical Neurophysiology guidelines [17, 18]. EP were conducted using Viking Quest equipment (Viasys Healthcare Inc., Conshohocken, Pennsylvania, USA).

Visual evoked potentials (VEP) were induced by a structural chess stimulus with alternating white and black fields emitted by a Nicolet Monitor (model NIC-1005), at a distance of 1 m. The angular size of individual squares was 1.1 degrees, and the whole field of view was 18 × 22 degrees. The left and right eyes were stimulated successively at a frequency of 1.88 Hz. The recording electrode was placed in the center line, on the occipital region (Oz), the reference electrode on the frontal region (Fz), and the ground electrode on the forearm. 75 responses were averaged in the frequency band 1-30 Hz at the analysis time of 500 ms. The latencies of N75, P100, N145 components, the relative P100 latency difference, and the P100-N145 amplitude were assessed. In patients with visual impairment, the examination was performed with corrective glasses.

Brainstem auditory evoked potentials (BAEP) were performed by stimulating the right and left ear with an acoustic stimulus (“click”) in rarefied polarity with a duration of 0.1 ms, at a presentation rate of 20.3 clicks/second and an intensity of 65 dB above the individually marked hearing threshold. The unexamined ear was masked with a noise of 35 dB above the hearing threshold. Responses were recorded identically using electrodes placed on the earlobe, with the reference electrode on the top of the head (A1 or A2, with respect to Cz) and the ground electrode on the forearm. 2.000 responses were averaged in the frequency band 150-3000 Hz in 10-ms analysis time. We analyze the absolute latencies of waves I, III, and V; interpeak latencies I-III, III-V, and I-V; and amplitudes of waves I and V.

Prolonged interpeak latencies of waves I-III and/or III-V were considered as pathological when they were accompanied by the extension of the I-V interpeak latencies. Prolonged latency of wave I was seen as abnormal when accompanied by changes in the latency of the subsequent auditory response. The range of mean values ±2 SD was assumed as being correct for individual BAEP and VEP components. A difference of 50% for the P100/N145 components between the left and the right ear, and a simultaneous difference of over 50% for the I and V wave amplitudes obtained during the left and right ear stimulation was considered pathological. The standards for individual BAEP latencies and interlatencies adopted for our laboratory are presented in Table 1.

Statistical analysis was performed using the Statistica 11.0 PL software. The Levene test determines the distribution of quantitative variables. A test was used to compare quantitative variables between the two analyzed groups with normal distribution,

Student *T*-test, for others—Mann–Whitney *U*. For more groups, one-way analysis of variance (ANOVA) was used. The chi^2^ test with Yates correction was used to compare the qualitative variables. Pearson's correlation coefficient was used to correlate quantitative variables with normal distribution, for the others—Spearman's correlation coefficient.

All hypotheses were verified at the level of statistical significance *p* ≤ 0.05.

## 4. Results

There were no significant differences in age (analysis of variance; *p* = 0.13) and sex distribution (chi^2^ = 0.61, *p* = 0.82) between HT patients and the control group.

### 4.1. Endocrinological Examination and Laboratory Parameters Analysis

The disease duration ranged from 9 to 48 years, and the average disease duration was 47 months (for women 49 months and for men 16 months).

The mean value for TSH was 1.78 ± 1.18 UIU/ml (normal value 0.35-5.6 UIU/ml), fT3 2.95 ± 0.48 pg/ml (normal value 2.5-3.9 pg/ml), fT4 1.08 ± 0.25 ng/dl (normal value 0.61-1.12 ng/dl), anti-TG 132.99 ± 247 IU/ml, anty-TPO 493 ± 374 IU/ml (Table 2).

### 4.2. Neurological and Laryngological Examination

The neurological examination was normal in all patients (100%). In 31 patients (31%), there were symptoms of increased neurovegetative excitability, such as tremors of eyelids and/or hands, positive Chvostek and Trousseau signs, increased dermographism, and hyperhidrosis of hands. The results of the MoCA and CDT were normal in all patients. None of the HT patients have abnormalities on brain magnetic resonance imaging. Laryngologial examination, the otoscopy, and audiogram were normal. All patients had type A tympanometric curves.

### 4.3. Comparison of Evoked Potentials between HT Patients and Control Group

**Figure 1:**
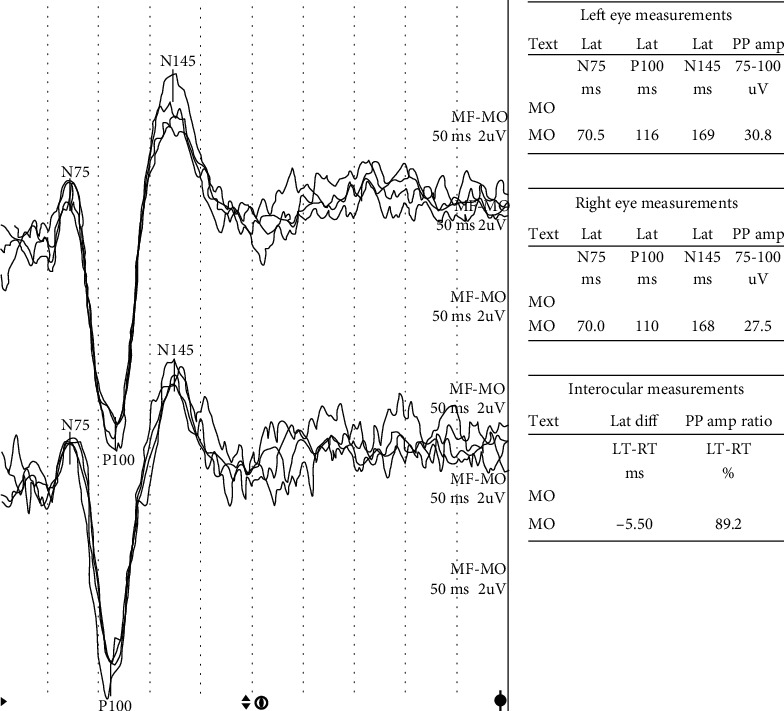
VEP in HT female—very high P100/N145 amplitude (L-30,8 *μ*V, R-27,5 *μ*V).

In the group of patients with Hashimoto's disease, compared to the control group, significant differences were obtained in the mean latency of P100 and N145 waves, and relative latency of P100 and amplitude P100/N145 (Table 3). In the group of patients, the average latencies P100, N145, and the average relative latency P100 were significantly longer, and the average amplitude of P100/N145 was significantly higher (*p* < 0.001) (Figure 1). A trend toward longer latency of the N75 component was also observed (*p* = 0.07).

Abnormal VEP was found in 25 patients (25%). Bilateral prolongation of absolute latency in the P100 wave was demonstrated in 19 cases, unilateral in 5 cases. Pathologically long relative latency was recorded in 11 people. None of the patients had an abnormal VEP amplitude.

In the group of patients compared to the control group, significantly longer mean V component latencies and III-V and I-V BAEP interpeak latencies (*p* < 0.001) were found, as well as significantly lower mean amplitudes of I and V BAEP waves (*p* < 0.001) (Table 4).

**Figure 2:**
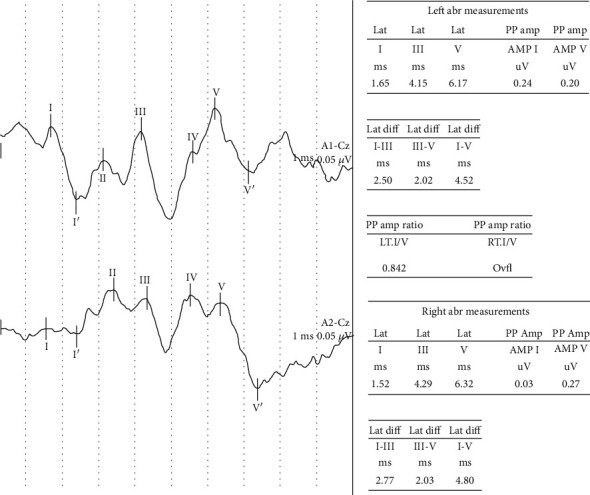
BAEP in HT male—prolonged latencies and interpeak latencies: III (L-4,15 ms, R-4,29 ms), V (L-6.17 ms, R-6,32 ms), I-III (L-2,5 ms, R-2,77 ms) and I-V (L-4,52 ms, R-4,8 ms).

Abnormal BAEP was registered in 14 (14%) patients. Six patients (6%) had an increase in V wave latency, including three wave I and III. Pathologically, long I-III interpeak latencies were demonstrated in 4 cases (in 2 unilaterally, in 2 bilaterally), III-V in 2 cases (in 1 unilaterally and in 1 bilaterally), and IV in 10 cases (in 4 unilaterally, in 6 on both sides) (Figure 2). A simultaneous over 50% difference in the amplitude of waves I and V obtained by stimulation of the right and left ear was found in 3 patients (3%). In 1 case, BAEP changes concerned both latency and amplitude, and in 2 cases only amplitude.

In total, abnormal VEP and BAEP parameters were found in 5 patients (5%), while abnormal VEP or BAEP parameters were found in 34 patients (34%).

### 4.4. Evoked Potentials Depending on the Length of the Disease

There were no statistically significant changes in VEP and BAEP parameters, depending on the length of the disease. Abnormal VEP and BAEP occurred similarly frequently at all time intervals of disease duration.

### 4.5. Comparison of Evoked Potentials Parameters between HT Patients and Control Group according to Age

Both in the group of patients and the control group of healthy people, longer latencies of VEP components were observed in the elderly (Table 5). In the group of patients, irrespective of age, a significantly longer mean absolute latency of the P100 wave was found (*p* < 0.001). The higher average P100/N145 amplitude in the youngest and oldest groups achieved statistical significance compared to the control group (*p* < 0.001). A longer average N145 wave latency was also observed in the group of patients over 40 (*p* = 0.01) and 60 years of age (*p* < 0.001).

Analyzing the correlation of latency values with the age of respondents, a more significant increase in latency over time was found in patients with Hashimoto's disease (P100 *p* = 0.0003; N145 *p* = 0.01) than in healthy people (P100 *p* = 0.02).

In the group of patients and the control group, the increase in age, latency, and interpeak latencies of individual BAEP components, as well as the decrease in I and V wave amplitude, was observed with age (Table 6). In the youngest group of patients (20-39 years of age), compared to the appropriate age control group, a significantly longer wave III latency, I-III interpeak latencies, and reduced I wave amplitude were found (*p* = 0.01). In the middle-aged (40-59 years of age) and the oldest (over 60 years of age) group of patients, significantly longer average V-wave latencies and average III-V and I-V interpeak latencies were demonstrated. In these groups, a lower average wave amplitude I and V was also found (*p* < 0.001).

The increase in latency of individual BAEP components over time (correlation of latency with the age of the subjects) was significant in patients with Hashimoto's disease compared to the control group.

### 4.6. Evoked Potentials in Patients with Symptoms of Increased Neurovegetative Excitability

In the group of patients with features of increased neurovegetative excitability compared to the group of patients without such features, a significantly shorter average P100 wave latency (*p* < 0.001) and a significantly higher P100/N145 wave amplitude (*p* < 0.01) were found (Table 7). Nevertheless, the average P100 latency in this subgroup was significantly longer compared to the latency obtained in the healthy control group (*p* = 0.01).

Analyzing BAEP, a significantly shorter average latency of wave III (*p* = 0.001), wave V (*p* = 0.05), and mean I-III (*p* = 0.003) and I-V (*p* = 0.01) were found (Table 6). However, these values were significantly longer compared to the corresponding data obtained in the healthy control group (*p* = 0.05).

### 4.7. Correlations of EP with Hormonal Concentrations, Anti-TG, and Anti-TPO Levels in HT Patients

There were no statistically significant correlations between the mean values of VEP parameters and the level of TSH, free thyroid hormones (fT3, fT4), antithyroid antibodies (anti-TG and anti-TPO), and the dose of levothyroxine used.

**Figure 3:**
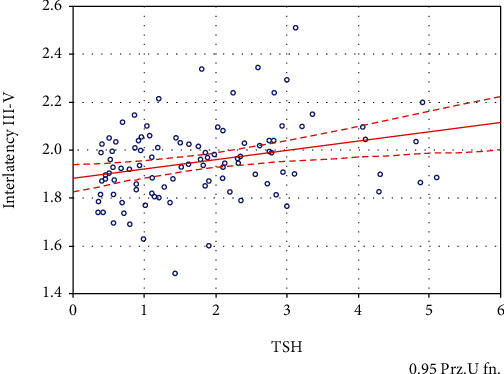
Correlation of III-V wave BAEP with TSH concentrations.

In the group of patients, a relationship between TSH level and III-V interpeak latency BAEP interaction was observed (Figure 3). This interpretation increased with the increase in TSH (R − Spearmann = 0.30, *p* < 0.001). Other BAEP parameters did not correlate with the TSH level. There was also no correlation between BAEP and the level of free thyroid hormones (fT3, fT4), antithyroid antibodies (anti-TG and anti-TPO), and the dose of levothyroxine used.

## 5. Discussion and Conclusion

Previous reports on both exogenous and endogenous EPs in patients with Hashimoto's disease were few and, usually, included case reports of a rare complication of Hashimoto's encephalopathy [19, 20]. In patients with hypothyroidism, Huang et al. [21] demonstrated a significantly longer P100 wave latency and a lower P100/N145 wave amplitude. In the group of hyperthyroiditis patients, they found a tendency to longer P100 latency, but without significant changes in amplitude. A significant limitation of the work of Huang et al. was a small group of subjects consisting of 16 people with hypothyroidism and 27 with hyperthyroidism. Khedr et al. [22] showed in the group of 23 patients with hypothyroidism an abnormal VEP in 52% of cases and a significantly longer latency of the P100 wave compared to the control group of healthy people. They did not observe changes in visual response amplitude. Nazliel et al. [23], comparing VEP in 24 patients with subclinical and in 24 patients with overt hypothyroidism, did not find significant changes in mean values of potential parameters in both groups. Abnormal VEP was recorded in 12.5% of patients regardless of the severity of hypothyroidism.

In the available literature, we found only a single study of Indian authors from 1996, who, analyzing VEP in 89 patients with various thyroid dysfunctions, also included patients with balanced hormonal function of this gland [24]. The evaluated group consisted of 28 people with hypothyroidism, 32 with hyperthyroidism, and 29 with autoimmune thyroiditis at the euthyroid stage. The authors showed a significantly longer P100 wave latency in each patient subgroup compared to the healthy controls. In people with thyroiditis and euthyroid disease, this latency was 109.00 ms vs. 101.08 ms, respectively, which was comparable to our results (106.89 ms vs. 100.65 ms). Contrary to our analyzes, Indian researchers obtained a significantly lower average P100/N145 wave amplitude in patients with thyroiditis and euthyroid disease compared to healthy individuals (2.41 *μ*V vs. 4.59 *μ*V).

The significantly higher amplitude of the P100 VEP wave found by us compared to the control group (12.35 *μ*V vs. 9.35 *μ*V) may indicate increased activity of the cerebral cortex in the examined patients. A similar phenomenon has been reported in people with epilepsy, migraine, or alcohol withdrawal syndrome, where excessive bioelectrical activity of the cortex is known [25, 26]. Similarly, in some cases of multiple sclerosis, EEG paroxysmal discharges of slow and convulsive potential against the background of normal or abnormal basic activity were recorded [27].

In these cases, tendencies to increase in amplitude and increase in VEP latency were observed. Epilepsy is more frequently observed in autoimmune diseases than in the general population [28]. This confirms the existence of hyperactivity of the cerebral cortex in patients with immune disorders. Seizures are part of the clinical picture of systemic lupus erythematosus, sarcoidosis, Sjögren's syndrome, celiac disease, Behcet's disease, Wegener's granulomatosis, and Hashimoto's disease [28, 29]. The pathogenesis of these seizures remains unclear. Among the possible causes, autoimmune mechanisms directly affecting the CNS structures (antibodies, autoimmune complexes, and proinflammatory cytokines), alteration of cerebral vascular structures during the inflammatory process, metabolic disorders, or treatment complications are considered [28, 29]. The first concept regarding CNS disease associated with antibodies appeared in the 1960s and was written by Lord Brain, who described a patient with EH [30]. It has been shown that in both children and in adults diagnosed with epilepsy, the presence of various antibodies is more frequent than in the general population [31]. Tsai et al. [32] found 28.3% of patients with idiopathic epilepsy to have elevated titers of antinuclear (ANA), antimicrosomal (AMA), or anti-TPO autoantibodies.

Frequently observed sensorineural hearing loss has resulted in the special interest of otolaryngologists in the study of BAEP in thyroid disorders resulting from immune system disorders [33]. However, as in the case of VEP, BAEP tests were performed mainly in patients with hormonal disturbances [21, 34]. Only Gawron et al. [19] analyzed BAEP in 30 children with Hashimoto's disease who are in an euthyroid state. Children with hearing loss were excluded from the study. In the group of patients, compared to healthy patients, significant longer wave I latency and III-V interlatencies were demonstrated. I also found a prolonged III-V interlatency, along with longer V-wave latency and I-V interlatency. Among the patients I examined in two, the latency of wave I was pathologically long. In these cases, changes in the BAEP component I may be a reflection of subclinical damage to the auditory nerve, in which Hashimoto's disease has an autoimmune basis [33]. In patients with overt or subclinical hypothyroidism, BAEP literature data are inconclusive or even contradictory. Some researchers have shown an increase in peripheral latency (I wave) and central BAEP components in hypothyroidism [34, 35]. Others have not confirmed such irregularities [36, 37].

Changes in VEP and BAEP observed in patients with decompensated thyroid function may be a consequence of hormonal disorders that affect peripheral function and the central nervous system. In patients with euthyroid disease, without clinical damage to the optic and auditory nerves and without features of a central neurological deficit, other causes of the abnormality should be sought. It is possible that the basis for changes in brain bioelectrical activity may be the negative impact of autoimmune mechanisms. Gawron et al. explained BAEP abnormalities with a subclinical form of thyroid encephalopathy [19].

Particularly, the large group of patients with tetanus (31%) is noteworthy. In the EP analysis, they were noticed by significantly shorter (though longer than in the control group) latencies of P100 VEP wave, III, V waves, and I-V BAEP interrelations as well as significantly higher P100/N145 wave amplitude. The observed changes in EP parameters may indicate an increase in neuronal excitability in the CNS. Thyroid diseases are listed as risk factors for tetany. This is especially true in cases requiring surgical treatment, a complication of which is damage or even removal of the parathyroid glands [38, 39]. Hashimoto's disease can coexist with hypoparathyroidism, especially when it has an autoimmune background. In people who have not undergone a strumectomy and who do not have hypoparathyroidism (as in the case of the patients examined by us), the etiology of the coexistence of Hashimoto's disease and tetany is unclear. A common factor in both these conditions may be vitamin D deficiency, which has a negative effect on the absorption of magnesium [1, 3]. The tetany, which is typically associated with electrolyte disturbances (hypomagnesaemia, hypocalcaemia), may accompany anxiety, neurotic disorders, hysteria, and emotional hyperactivity [39]. In the available literature, however, we did not find information as to how often Hashimoto's disease is accompanied by tetany symptoms or features of increased neurovegetative excitability.

Symptoms of tetany and increased neurovegetative excitability often coexist with hyperventilation incidents and calcium disorders. The effects of hyperventilation on cerebral excitability and synaptic transmission have been demonstrated in both animal and human studies. In experimental works, during hyperventilation of rats, increased bioelectric excitability of neurons in the hippocampal region was observed [40]. In human studies using transcranial magnetic stimulation (TMS), after hyperventilation periods, an increase in amplitude and shortening of motor potential latency (MEP) were found [41]. This phenomenon has been associated with a secondary reduction in carbon dioxide partial pressure and a decrease in ionized calcium in vascular smooth muscle cells. This condition results in a contraction of these muscles and a decrease in regional cerebral flow. In turn, impaired flow causes an increase in anaerobic metabolism, followed by an increase in the release of glutamate, a neurotransmitter that increases neuronal excitability. Hypoxia in these conditions is an additional factor increasing neuron sensitivity to glutamate [42, 43].

Statistical analysis of individual EP parameters showed a positive correlation between TSH values and III-V BAEP interaction. As the level of TSH increased, this time increased, which indicates a disturbance of auditory conduction in the upper part of the brainstem in patients with higher thyrotropin levels. No similar results were found in the available literature.

Our observations confirm the fact of brain bioelectrical activity disturbances in HT patients who did not have central nervous system deficits. The increased amplitude of the VEP in the course of HT may indicate increased cerebral cortex activity. Disorders of brain bioelectrical activity in the course of HT may be associated with an ongoing autoimmune process. The analysis of the VEP and BAEP may be useful in assessing brain bioelectrical activity in the course of HT [44].

## Figures and Tables

**Table 1:** Normative parameters for BAEP and x + 2 SD. SD: standard deviation.

BAEP	Normal value (ms)	*x* + 2 SD
Latency (ms)		
I	1.81 ± 0.11	2.01
III	3.90 ± 0.17	4.29
V	5.82 ± 0.28	6.20
I-III	2.12 ± 0.19	2.75
III-V	1.89 ± 0.20	2.51
I-V	4.05 ± 0.28	4.95

**Table 2:** Clinical characteristics of HT patients.

	*n* = 100	Women (*n* = 92)	Men (*n* = 8)
TSH (UIU/ml)	1.78 ± 1.18	1.72 ± 1.1	2.54 ± 1.71
fT3 (PG/ml)	2.95 ± 0.48	2.93 ± 0.47	3.14 ± 0.6
fT4 (NG/dl)	1.08 ± 0.25	1.08 ± 0.23	1.08 ± 0.42
Anty-TG (IU/ml)	132.99 ± 247	121.7 ± 238.5	262.5 ± 324
Anty-TPO (IU/ml)	493 ± 374	517 ± 370	221.7 ± 323
Dose of levothyroxine (*μ*g/day)	64 ± 37	63 ± 36	69 ± 45

**Table 3:** Mean values of the latency (ms) and amplitude (*μ*V) of VEP parameters in HT patients and in the control group.

VEP	HT patients *n* = 100			Control group *n* = 50			*p*
	Mean	Median	SD	Mean	Median	SD	
Latency (ms)							
N75	71.81	71.00	6.36	70.25	69.50	5.45	0.07
P100	106.89	105.00	7.78	100.65	100.00	3.98	*<0.001*
N145	149.31	148.50	16.33	140.65	143.00	11.44	*<0.001*
P100 Lo-Po	2.91	2.00	2.63	1.58	1.00	1.45	*<0.001*
Amplitude (*μ*V)							
P100/N145	12.35	11.45	5.24	9.35	9.20	3.13	*<0.001*

**Table 4:** Mean values of the latency (ms) and amplitude (*μ*V) of BAEP parameters in HT patients and in the control group.

BAEP	HT patients n =100			Control group n =50			*p*
	Mean	Median	SD	Mean	Median	SD	
Latency (ms)							
I	1.70	1.69	0.14	1.66	1.67	0.14	0.1
III	3.85	3.83	0.19	3.81	3.83	0.14	0.29
V	5.80	5.80	0.28	5.67	5.68	0.20	*<0.001*
I-III	2.15	2.14	0.19	2.15	2.16	0.12	0.91
III-V	1.95	1.95	0.19	1.86	1.83	0.17	*<0.001*
I-V	4.10	4.10	0.28	3.98	4.00	0.24	*<0.001*
Amplitude (*μ*V)							
I	0.21	0.19	0.12	0.30	0.30	0.11	*<0.001*
V	0.38	0.37	0.13	0.46	0.46	0.17	*<0.001*

**Table 5:** Mean values of the latency (ms) and amplitude (*μ*V) of VEP parameters in HT patients and in the control group depending on the age.

VEP	HT patients *n* = 100			Control group *n* = 50			*p*
	Mean	Median	SD	Mean	Median	SD	
Age	20-39 y.						
	*n* = 33		*n* = 18				
Latency (ms)							
N75	70.58	70.00	4.63	69.58	69.50	4.28	0.35
P100	103.82	103.00	4.77	99.78	100.00	2.72	*<0.001*
N145	145.53	147.00	13.13	140.03	144.50	13.22	0.08
P100 lo-Po	2.39	2.00	2.02	1.83	2.00	1.14	0.73
Amplitude (*μ*V)							
P100/N145	12.31	12.20	4.64	9.02	9.02	2.70	*<0.001*
Age	40-59 y.						
	*n* = 45		*n* = 21				
Latency (ms)							
N75	72.31	71.50	6.82	69.69	69.00	6.74	0.02
P100	107.33	105.00	9.13	99.61	99.25	4.33	*<0.001*
N145	148.47	147.00	17.95	139.79	142.00	12.06	*0.01*
P100 Lo-Po	3.15	3.00	2.1	1.29	1.00	1.05	*<0.001*
Amplitude (*μ*V)							
P100/N145	11.66	11.20	4.79	10.27	10.20	3.42	0.12
Age	60 y. and more						
	*n* = 22		*n* = 11				
Latency (ms)							
N75	72.60	70.75	7.37	72.43	71.75	3.77	0.58
P100	110.33	110.00	6.99	104.1	103.00	3.20	*<0.001*
N145	156.00	153.50	15.51	143.32	144.00	5.66	*<0.001*
P100 Lo-Po	3.19	2.00	3.33	1.73	1.00	1.79	0.31
Amplitude (*μ*V)							
P100/N145	13.66	11.90	6.49	8.16	7.79	2.83	*<0.001*

**Table 6:** Mean values of the latency (ms) and amplitude (*μ*V) of BAEP parameters in HT patients and in the control group according to age.

BAEP	HT patients *n* = 100			Control group *n* = 50			*p*
	Mean	Median	SD	Mean	Median	SD	
Age	20-39 y.						
	*n* = 33			*n* = 18			
Latency (ms)							
I	1.69	1.65	0.13	1.66	1.65	0.13	0.28
III	3.77	3.74	0.17	3.83	3.84	0.13	*0.01*
V	5.66	5.64	0.21	5.67	5.66	0.23	0.76
I-III	2.08	2.05	0.17	2.16	2.15	0.14	*0.01*
III-V	1.89	1.89	0.17	1.84	1.81	0.18	0.06
I-V	3.96	3.93	0.25	3.93	3.99	0.35	0.77
Amplitude (*μ*V)							
I	0.24	0.20	0.14	0.31	0.31	0.10	*<0.001*
V	0.40	0.40	0.12	0.49	0.46	0.21	0.76
Age	40-59 y.						
	*n* = 45			*n* = 21			
Latency (ms)							
I	1.72	1.73	0.13	1.67	1.67	0.15	0.09
III	3.87	3.85	0.18	3.82	3.83	0.14	0.21
V	5.84	5.85	0.22	5.71	5.75	0.19	*<0.001*
I-III	2.15	2.15	0.20	2.15	2.15	0.10	0.95
III-V	1.97	1.94	0.18	1.88	1.86	0.18	*<0.001*
I-V	4.12	4.13	0.25	4.04	4.02	0.17	*0.05*
Amplitude (*μ*V)							
I	0.21	0.19	0.12	0.27	0.25	0.10	*<0.001*
V	0.36	0.35	0.14	0.41	0.40	0.14	*0.01*
Age	60 y. and more						
	*n* = 22			*n* = 11			
Latency (ms)							
I	1.72	1.70	0.13	1.67	1.68	0.15	0.09
III	3.93	3.95	0.22	3.75	3.73	0.16	*<0.001*
V	5.92	5.94	0.37	5.71	5.59	0.16	*<0.001*
I-III	2.15	2.27	0.20	2.15	0.10	0.16	0.95
III-V	2.00	1.99	0.22	1.85	1.82	0.15	*<0.001*
I-V	4.24	4.25	0.29	3.97	3.98	0.12	*<0.001*
Amplitude (*μ*V)							
I	0.18	0.19	0.10	0.27	0.34	0.11	*<0.001*
V	0.36	0.34	0.12	0.40	0.50	0.11	*<0.001*

**Table 7:** Significant changes in VEP and BAEP parameters depending on increased neurovegetative excitability in patients with HT.

VEP	Increased neurovegetative excitability						*p*
	Presence *n* = 31			Absence *n* = 69			
	Mean	Median	SD	Mean	Median	SD	
Latency (ms)							
P100	104.66	104.00	4.98	107.89	105.50	8.58	*<0.001*
Amplitude (*μ*V)							
P100/N145	13.49	12.60	4.75	11.48	10.75	5.13	*<0.01*
BAEP							
Latency (ms)							
III	3.79	3.70	0.22	3.87	3.85	0.19	*0.001*
V	5.73	5.70	0.22	5.83	5.83	0.29	*0.05*
I-III	2.09	2.05	0.20	2.17	2.17	0.18	*0.003*
I-V	4.03	4.04	0.26	4.13	4.12	0.27	*0.01*

## Data Availability

The numerical data used to support the findings of this study are available from the first and corresponding author upon request.
